# Virtual Reality for Sedation During Atrial Fibrillation Ablation in Clinical Practice: Observational Study

**DOI:** 10.2196/26349

**Published:** 2021-05-27

**Authors:** Thomas Roxburgh, Anthony Li, Charles Guenancia, Patrice Pernollet, Claire Bouleti, Benjamin Alos, Matthieu Gras, Thomas Kerforne, Denis Frasca, François Le Gal, Luc Christiaens, Bruno Degand, Rodrigue Garcia

**Affiliations:** 1 Department of Anesthesia and Critical Care University Hospital of Poitiers Poitiers France; 2 Cardiology Clinical Academic Group St George's, University of London London United Kingdom; 3 Cardiology Department University Hospital of Dijon Dijon France

**Keywords:** connected devices, virtual reality, atrial fibrillation, pain management

## Abstract

**Background:**

Connected devices are dramatically changing many aspects in health care. One such device, the virtual reality (VR) headset, has recently been shown to improve analgesia in a small sample of patients undergoing transcatheter aortic valve implantation.

**Objective:**

We aimed to investigate the feasibility and effectiveness of VR in patients undergoing atrial fibrillation (AF) ablation under conscious sedation.

**Methods:**

All patients who underwent an AF ablation with VR from March to May 2020 were included. Patients were compared to a consecutive cohort of patients who underwent AF ablation in the 3 months prior to the study. Primary efficacy was assessed by using a visual analog scale, summarizing the overall pain experienced during the ablation.

**Results:**

The AF cryoablation procedure with VR was performed for 48 patients (mean age 63.0, SD 10.9 years; n=16, 33.3% females). No patient refused to use the device, although 14.6% (n=7) terminated the VR session prematurely. Preparation of the VR headset took on average 78 (SD 13) seconds. Compared to the control group, the mean perceived pain, assessed with the visual analog scale, was lower in the VR group (3.5 [SD 1.5] vs 4.3 [SD 1.6]; *P*=.004), and comfort was higher in the VR group (7.5 [SD 1.6] vs 6.8 [SD 1.7]; *P*=.03). On the other hand, morphine consumption was not different between the groups. Lastly, complications, as well as procedure and fluoroscopy duration, were not different between the two groups.

**Conclusions:**

We found that VR was associated with a reduction in the perception of pain in patients undergoing AF ablation under conscious sedation. Our findings demonstrate that VR can be easily incorporated into the standard ablation workflow.

## Introduction

Atrial fibrillation (AF) ablation is an established therapy for patients with symptomatic AF [1]. The number of AF ablations is increasing year on year, but the availability of general anesthesia does not meet demand [2]. As such, it is now commonplace for procedures to be performed under conscious sedation. However, patients’ pain and discomfort during AF ablation may be associated with poorer outcomes [3].

Connected devices are dramatically changing many aspects in health care [4-7]. While in cardiology, the majority of devices are intended to monitor heart rhythm, others have a therapeutic purpose [8-10]. One such device, the virtual reality headset, is the subject of numerous studies [11-15] and has recently shown to improve analgesia in a small sample of patients undergoing transcatheter aortic valve implantation [16,17]. To our knowledge, this device has not been tested in other cardiac procedures.

We aimed to investigate the feasibility and effectiveness of VR in patients undergoing catheter ablation of AF under conscious sedation.

## Methods

### Recruitment

From March to May 2020, all consecutive patients in whom an AF ablation was performed using cryoballoon (Arctic Front Advance; Medtronic) at the University Hospital of Poitiers were included. All participants received VR using a Deepsen headset on top of the usual analgesia protocol. The VR technique uses cognitive saturation in association with cardiac coherence breathing, music therapy, and gamification [18]. At the beginning of the session, 5 minutes of cardiac coherence breathing was delivered. Then, the patient was immersed in 1 of 5 3D computer-simulated scenarios. During the whole procedure, music therapy and gamification were also used. The patient played an active role as he or she interacted with the virtual environment, which aimed to deepen the immersion to unconsciously disconnect the patient from painful moments in the procedure. Our analgesia protocol consisted of 1 g of intravenous (IV) paracetamol, 20 mg of IV nefopam, 1 mg of IV midazolam, and 3 mg of IV morphine just before the start of the procedure. Patients could also request additional analgesia, in which case further boluses of 1 mg of morphine were given. Patients who underwent AF ablation with VR were compared to a consecutive cohort of patients who received routine AF cryoablation in the 3 months prior to the study using the standard analgesia protocol.

### Outcomes

The feasibility of VR was assessed by the number of patients who refused this technique, the tolerance of the VR headset, and the time taken to install the device. Primary efficacy was assessed by using a visual analog scale (VAS), shown to the patient 45 minutes post procedure when they were asked to select a single point on the scale to summarize the overall pain experienced during the ablation [19]. The maximum pain intensity perceived was also recorded using a VAS score. Finally, the patient’s comfort was assessed using a numerical scale, with 0 being “the most uncomfortable procedure you could have” and 10 being “the most comfortable procedure you could have.” Oral informed consent was obtained from all participants. According to French legislation, this study was declared to the Commission Informatique et Libertés and did not require the approval of an Ethics Committee as this device is CE marked and is already used in routine clinical practice in some centers in France.

### Statistical Analysis

Continuous variables were expressed as mean (SD) and categorical variables were presented as numbers and percentages. Comparisons between groups were performed using the Student *t* test or the Mann-Whitney *U* test for continuous variables as appropriate, and the chi-square test for categorical variables. Analyses were performed using SPSS 22 (IBM Corp) statistical software.

## Results

### Feasibility

**Figure 1:**
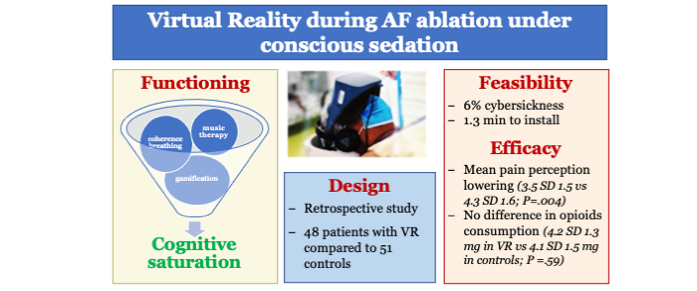
Virtual reality (VR) use during atrial fibrillation (AF) ablation.

A total of 48 patients were enrolled to receive VR during AF cryoablation procedure (mean age 63.0, SD 10.9 years; n=16, 33.3% females). No patient refused to use the device, although 7 (14.6%) terminated the VR session prematurely. Four patients had a vasovagal reaction and 3 experienced cybersickness (vertigo: n=2; headache: n=1) (Figure 1). Preparation of the VR headset took on average 78 (SD 13) seconds.

### Efficacy

The control group comprised 51 patients. No significant differences were found between control and intervention groups in terms of age, gender, New York Heart Association functional class, left ventricular ejection fraction, or previous medications (Table 1).

**Table 1:** Baseline characteristics.

Characteristic		Virtual reality group (n=48)	Control group (n=51)	*P* value
Age (years), mean (SD)		63.0 (10.9)	64.5 (10.4)	.46
Gender (male), n (%)		32 (67)	39 (76)	.28
BMI (kg/m^2^), mean (SD)		28.0 (4.6)	27.4 (4.9)	.55
**NYHA functional class^a^, n (%)**				.15
	Class I-II	30 (83)	33 (89)	
	Class III-IV	6 (17)	4 (11)	
Hypertension, n (%)		19 (40)	17 (53)	.18
Diabetes mellitus, n (%)		5 (10)	2(3)	.21
**Atrial fibrillation, n (%)**				.23
	Paroxysmal	19 (40)	27 (53)	
	Persistent	29 (60)	24 (47)	
	Long-standing persistent	0 (0)	0 (0)	
**CHA_2_DS_2_-VASc^b^ score (SD)**				.66
	0-1	15 (32)	15 (29)	
	2-3	29 (60)	29 (57)	
	≥4	4 (8)	7 (14)	
Ischemic cardiomyopathy, n (%)		4 (8)	11 (22)	.06
Systolic blood pressure (mmHg), mean (SD)		137 (18)	136 (23)	.77
**Electrocardiogram**				
	Heart rate (bpm), mean (SD)	72 (19)	74 (22)	.63
	Sinus rhythm, n (%)	30 (63)	35 (69)	.79
**Echocardiography, mean (SD)**				
	Left ventricular ejection fraction (%)	59 (9)	59 (10)	.74
	Left atrial volume (mL/m^2^)	76 (28)	70 (23)	.18
NT-proBNP^c^ (ng/L), mean (SD)		656 (956)	760 (1127)	.25
**Medication, n (%)**				
	Anticoagulant	47 (98)	50 (98)	.72
	Beta-blockers	44 (92)	46 (90)	.80
	Angiotensin-converting enzyme inhibitor	23 (48)	22 (43)	.63
	Antiplatelet agents	2 (4)	5 (10)	.23
	Amiodarone	19 (40)	29 (57)	.36

^a^A New York Heart Association (NYHA) functional class was calculated for patients with heart failure (n=36 in the virtual reality group and n=37 in the control group).

^b^CHA_2_DS_2_-VASc: congestive heart failure, hypertension, age ≥75 years, diabetes mellitus, prior stroke or transient ischemic attack or thromboembolism, vascular disease, age 65-74 years, sex category.

^c^NT-proBNP: N-terminal pro B-type natriuretic peptide.

**Figure 2:**
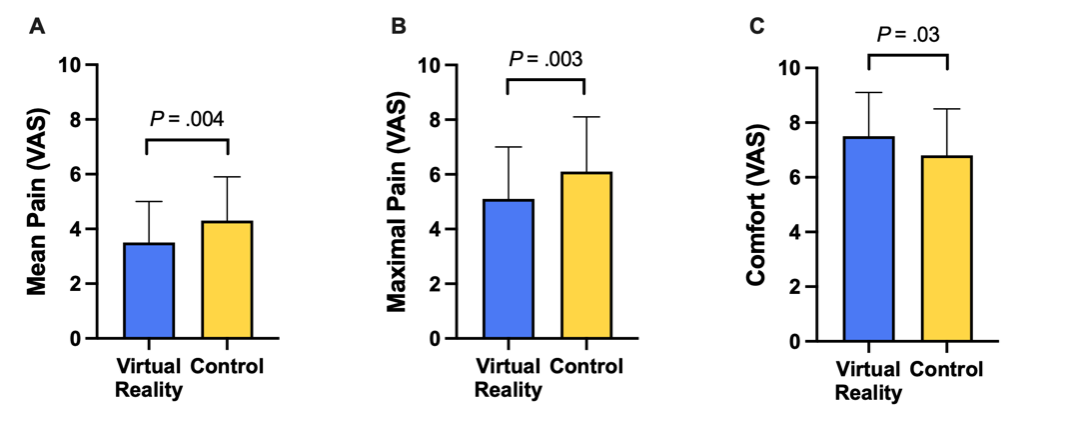
Mean pain, maximal pain, and comfort assessment in the virtual reality and control groups.

Mean and maximal perceived pain were lower in the VR group (mean pain: 3.5 [SD 1.5] vs 4.3 [SD 1.6]; *P*=.004; maximal pain: 5.1 [SD 1.9] vs 6.1 [SD 2.0]; *P*=.003) and comfort was higher in the VR group (7.5 [SD 1.6] vs 6.8 [SD 1.7]; *P*=.03) (Figure 2). On the other hand, morphine consumption was not different across the groups (VR: 4.2 [SD 1.3] mg vs control: 4.1 [SD 1.5] mg; *P*=.59). In addition, procedure and fluoroscopy duration were not different between the two groups. Lastly, 3 (6.3%) patients had transient phrenic palsy in the VR group vs 6 (11.8%) in the control group (*P*=.34) and vagal reaction occurred in 4 (8.3%) patients in the VR group vs 2 (3.9%) in the control group (*P*=.60).

## Discussion

### Principal Findings

To our knowledge, this study is the first to evaluate the use of immersive VR to improve pain perception during cryoballoon AF ablation. Less than 10% of patients experienced cybersickness, and the device was set up in under 2 minutes. Compared to the control group, VR during AF ablation was associated with pain reduction and comfort improvement but did not lead to a reduction in opioid consumption.

### Effect of VR During the AF Ablation Procedure

Several studies have assessed the feasibility and/or effectiveness of VR in different clinical settings [20-22]. However, the number of patients included in these studies was small. The present study showed that in patients undergoing AF ablation, VR uptake was high and well tolerated, suggesting that it can be widely adopted during AF ablation procedures performed under conscious sedation. Moreover, setup can be carried out by the operator or the nurse, and increased the total duration of the procedure by less than 2% [23], which is acceptable, as the device was associated with lower pain with the same level of opioid consumption. It can be speculated whether the absence of a placebo in the control group could have influenced the results. However, we could argue that the placebo effect of VR is still valid because it is really this subjective assessment of pain reduction and comfort increase that are the endpoints we are trying to achieve. Nevertheless, the reduction in pain perception seen in this study did not result in reduced opioid use since morphine consumption was not different between the two groups. This lack of difference might be related to the low level of additional opioids required after the initial bolus of morphine. Indeed, only a mean of 1.2 mg and 1.1 mg were needed on top of the initial bolus in the VR and control groups, respectively. Overall, our results are in line with the literature [24]. In a recent systematic review, Smith et al [24] suggested that VR was effective for analgesia in a variety of different clinical settings but could also have disadvantages.

### Advantages and Drawbacks of VR

Other alternative techniques such as music therapy and hypnosis have been developed to relieve pain during conscious sedation anesthesia, but each have their drawbacks and advantages [25,26]. As the brain can only process a limited amount of information, mind saturation using VR aims to increase nonpainful input and limit the transmission of pain information according to the gate control theory [27]. Moreover, contrary to hypnosis, VR has a minimal learning curve and does not require specialist training, which may facilitate widespread adoption by other health care centers [19]. On the other hand, VR may elicit unpleasant reactions, such as cybersickness, in patients prone to vertigo or seasickness caused by conflicting sensory signals [28]. While the patient receives visual signals informing him or her that he or she is moving, no corroborating information is provided by the vestibular organs. Cybersickness has been described to occur in 20% to 80% of cases [29], although it occurred in only 18.8% of the sample in Bruno et al’s [17] study. In our study, the occurrence of this side effect was even lower (6.3%) and may be due to younger age, fewer comorbidities, and better hemodynamic stability in patients undergoing AF ablation compared to those who underwent the transcatheter aortic valve implantation procedure.

### Limitations

This study was a nonrandomized, single-center study. Nevertheless, the relatively high number of patients in each group and consecutive inclusion have limited bias. Moreover, baseline characteristics were not different between groups. Finally, patients’ prior experience with interactive games may have influenced the effect of VR and its associated side effects, which was not systematically assessed or taken into account in our study.

### Conclusion

Our study demonstrates that VR can be easily incorporated into the standard AF ablation workflow. Further, it was associated with a reduction in the perception of pain, even if it did not result in less opioid consumption, and improved patient experience. Larger randomized studies are needed to confirm these promising findings.

## Abbreviations

AF: atrial fibrillation
IV: intravenous
VAS: visual analog scale
VR: virtual reality
